# Perspectives and Experiences of People Receiving Care on a De‐escalation Intervention to Reduce Restrictive Practices in Acute Mental Health Units

**DOI:** 10.1111/inm.70300

**Published:** 2026-06-29

**Authors:** Esario IV Daguman, Dane Owen, Jacqui Yoxall, Richard Lakeman, Marie Hutchinson

**Affiliations:** ^1^ Faculty of Health Southern Cross University Coffs Harbour New South Wales Australia; ^2^ Integrated Mental Health, Alcohol and Other Drugs Coffs Harbour Base Hospital Coffs Harbour New South Wales Australia; ^3^ Faculty of Health Southern Cross University Lismore New South Wales Australia; ^4^ School of Nursing and Midwifery University of Southern Queensland Toowoomba Queensland Australia

**Keywords:** coercion, intervention evaluation, mental health service, psychiatric hospitals, psychiatric nursing

## Abstract

Interventions aimed at reducing restrictive practices are also designed to enhance the service experience in acute mental health units. However, people with experience of coercive engagement with these services are seldom involved as active contributors in evaluative research on interventions to reduce restrictive practices. With the meaningful involvement of lived experience practitioners, this research was aimed at examining care recipients' service experiences and perspectives on nurses' therapeutic responses during the implementation of a de‐escalation intervention in three adult inpatient units within New South Wales, Australia, from March 2024 to April 2025. Nested within a larger study employing a mixed concurrent control design, this research evaluated the effectiveness and process of the *Safe Steps for De‐escalation* through comparisons of unmatched measures of empowerment, dehumanisation, and staff actions on violence prevention across three time points, as well as through a reflective thematic analysis of semi‐structured interviews. Safe Steps is a structured approach for therapeutic responding, targeting nurses' relationship‐promotion behaviours to increase focus on minimising the use of restrictive practices. Eighty‐six inpatients completed the unmatched measures, with nine participating in interviews following discharge. No significant changes were noted in quantitative measures over time. Five themes emerged from the qualitative analysis: (i) *Clarity calms; confusion harms*, (ii) *Control cuts deep*, (iii) *Systems strain; people break*, (iv) *Connection is treatment in itself,* and (v) *Meaning‐making outweighs medicine*. These findings cast acute inpatient units in a light akin to a power circuit, elevating the need to make inpatient admissions more reflective of everyday life outside the units.

## 1 Introduction

Improving people's^1^ experience of engaging with public acute mental health services through interventions aimed at reducing the use of restrictive practices involves being attuned to the lived experience of people receiving care. However, eliciting experiences of using services or the experience of ‘treatment’ is rare in intervention evaluation research in the context of public acute mental health services, with individuals who experienced coercive engagements often excluded as active researchers (Gooding et al. 2020). Even when people with experience of using mental health services are included, existing value and power structures and relationships endure. It has been said that research on the concept of de‐escalation, for instance, risks reinforcing the othering of people receiving care when it is framed as a set of staff‐driven techniques, rather than as a collaborative process that acknowledges the person's role in achieving a sense of safety (Price et al. 2024).

Understanding people's experiences of mental health services can be facilitated by drawing on implementation and evaluation frameworks that account for both the local and broader contexts (Husum et al. 2024). Within this approach, unexpected events are considered, including parts of an intervention that are not deployed as planned or that interact in unforeseen ways with each other and the context (Morgan‐Trimmer et al. 2021). These approaches help discern how interventions work and how evidence on their effectiveness can inform practice (Moore et al. 2015). However, in a systematic mapping of 150 non‐pharmacological interventions to reduce restrictive practices in adult mental health settings, theory‐informed interventions and methodologically‐rigorous evaluations were found to be rare (Baker et al. 2021).

### 1.1 Formal Coercion, Ward Climate and People's Experiences of Service Changes

Coercion is a form of othering. It is the restriction of a person's autonomy by overturning their own will (Faissner et al. 2025). It has been described as a ‘process of de‐subjectivation’ (Verbeke et al. 2019, 89), where individuals lose their sense of self and are dehumanised. In public acute mental health services, most admissions are increasingly involuntary (Sheridan Rains et al. 2019), which suggests that coercion is present from the outset. Within these services, coercion is further typified informally by restrictive ward rules and denial of people's requests, and formally by seclusion, physical restraint, and forced medication (Paradis‐Gagné et al. 2025). While formal coercion is sometimes justified as necessary to preserve life, its widespread use in psychiatry has been considered to reflect societal risk aversion, rather than evidence of therapeutic benefit (Galderisi et al. 2024). It is this second type of coercion, otherwise known as restrictive practices, that governments call to minimise and, if possible, eliminate (e.g., NSW Health 2017). As a result, much evaluative research has focused on more compassionate alternatives to restrictive practices, such as Safewards (Bowers et al. 2015), trauma‐informed approaches (Muskett 2014), and sensory rooms (Champagne and Sayer 2003).

Evidence for the impact of restrictive practice‐reduction interventions on the ward's social climate and people's experience of service changes is limited and mixed. A systematic review of 23 studies on improving ward social climate found that only three initiatives explicitly targeted reducing the use of restrictive practices (Dickens et al. 2022), with only one linked to both reduced restrictive practices and an improved ward atmosphere (Mistral et al. 2002). A mixed‐method systematic review of Safewards found that the Discharge Messages intervention, intended to share hope and advice from lived experience practitioners, was experienced positively in an Australian implementation when discharge rates were high; however, its impact was less pronounced in a UK iteration when discharge rates were low (Ward‐Stockham et al. 2022). In a systematic review and narrative synthesis of sensory rooms, the ability to self‐regulate in a calm environment was experienced as empowering by the individuals receiving care, while some participants reported feeling bored and anxious (Haig and Hallett 2022). In a scoping review on trauma‐informed approaches in various mental health settings, intended to increase clinicians' awareness of trauma and prevent re‐traumatisation, many participants reported being cared for, although some found being directly asked about their trauma as difficult, especially in a ‘trauma‐informed crisis house’ (Saunders et al. 2023).

### 1.2 The Safe Steps for De‐Escalation

The Safe Steps for De‐escalation, or simply the Safe Steps, was conceptualised in 2018 in one Australian acute mental health unit. At the time of its initial development, no de‐escalation model offered sufficient empirical evidence or practical guidance for nurses in high‐acuity settings (NSW Health MNCLHD 2020), particularly in Australia, where many nurses lack specialist mental health qualifications (Hurley et al. 2024). Existing programs gave little recognition to the real‐world capabilities required to distinguish and respond to various levels of emotional activation, troubling behaviours, and interpersonal conflict within acute mental health units. As a result, an intervention has been developed to support the cultivation of nurses' situational and contextual awareness (see Daguman et al. 2025 for evidence of the importance of this value).

Safe Steps centres on a four‐step approach for de‐escalation, designed to offer nurses the latitude to exercise their intuitive expertise. It was structured to support nurses in creating and maintaining a supportive space, where individuals in distress can explore their issues, feel genuinely heard, collaborate on next steps, and leave with a shared understanding of actions that may help sustain a sense of safety beyond the de‐escalation conversation. Training on the four‐step approach and its underlying values, along with restrictive practice review meetings, supported its implementation. There is evidence for the structure and progression in the use of the relational capabilities promoted in the Safe Steps (Daguman et al. 2025) and for the effectiveness of concurrently conducting restrictive practice review meetings, centred on nurses' practice of relational capabilities, in reducing the seclusion rate in an acute mental health inpatient unit (Daguman, Taylor, et al. 2025).

Given the initial success of the Safe Steps, it has been further refined and evaluated through a translational research project. Online supplementary resources, offered as part of the training on the four‐step approach, were co‐developed with two Aboriginal Elders, three Aboriginal health advocates, two Aboriginal peer workers, two lived experience practitioners, and two transcultural health experts. The content of these online resources and the restrictive practice reviews is summarised in Table 1.

**TABLE 1:** An overview of the intervention components of the Safe Steps.

Intervention component	Brief description
Four‐step De‐escalation Framework	A four‐step framework for recognising, responding to, and reviewing escalating situations in acute mental health units, developed by mental health clinicians. It offers nurses a phased approach to facilitate safe, supportive interactions in which the person in distress can have time to think and process their concerns, feel normalised and accepted, collaboratively brainstorm the next steps, and explore actions for self‐management beyond the immediate de‐escalation encounter.
In‐Person and Online Training	An educational, unit‐wide resource offering that introduced participants to the four‐step framework, which included in‐person sessions and self‐paced multimedia modules (text, diagrams, audio, video). Developed in collaboration with peer workers, Aboriginal Elders and health leads, and multidisciplinary staff, the modules addressed: (1) framework overview and reflection on culture, introduction of trauma and emotional dysregulation; (2) co‐de‐escalation and cultural safety; (3) emotional intelligence and trauma‐informed care‐related concepts; and (4) ways to apply the four steps in live situations.
Restrictive Practice Review Meetings	From the third month, monthly reviews were embedded into existing in‐service sessions that had nurse educators present aggregated de‐escalation log data, detailing context, behaviour functions, and strategies used to prompt reflection, celebrate nurse participants' practice of relational competencies, and explore incidences of restrictive practices and possible alternative ways to prevent them.

The current research was focused on understanding the perspectives of discharged inpatients about their experience of the acute mental health service, including nurses' therapeutic responses to emotional distress, troubling behaviours, and interpersonal conflict. The research was also intended to examine the impact of the Safe Steps implementation on unmatched measures of attitudes towards dehumanisation, empowerment, and staff actions related to violence prevention. It was hypothesised that these three standardised measures would improve after the intervention implementation, compared to the within‐group baseline (pre‐intervention) levels.

## 2 Methods

### 2.1 Design

A mixed concurrent control design was employed in the larger study in which this research was nested. Qualitative and quantitative data were gathered concurrently in separate phases of the larger study and were mainly integrated at the reporting and interpretation stages to examine *if* and *how* the Safe Steps works and what sustains its effective and competent implementation. Unmatched measures (through surveys) and interviews were undertaken with people receiving care in the intervention implementation sites to address the larger study's second and third objectives: i.e., to examine the impact of implementing Safe Steps on service experience and perceived staff actions on violence prevention, as well as to determine what factors influenced the achievement of successful intervention implementation from the perspectives and experiences of people receiving care. The remaining objectives of the larger study were addressed elsewhere (Daguman, Yoxall, et al. 2025; Daguman et al. 2026b). The implementation and evaluation framework, the mixed methods design diagram, and the intervention evaluation protocol are also available online (Daguman, Taylor, Flowers, Owen, et al. 2025). This research was reported in line with the Journal Article Reporting Standards for Mixed Methods (JARS–Mixed), following the Mixed Methods Article Reporting Standards (MMARS) to ensure integration of qualitative and quantitative data (Levitt et al. 2018).

A researcher‐made implementation and evaluation framework guided the larger study evaluating the Safe Steps. It had a fundamental orientation towards iterative approaches that enable the accounting for, and accommodation of, real‐world acute inpatient contexts and mental health nursing practice dynamics. This orientation was based on an overview of systematic reviews on restrictive practice‐reduction interventions, indicating that successful implementation and evaluation of interventions within the field will need to be carried out pragmatically, guided by properties of complex adaptive systems (Daguman et al. 2024).

Safe Steps was pragmatically implemented between March 2024 and April 2025, with four quarterly time points, in three adult inpatient units that operate 24/7 within three public hospitals in New South Wales (NSW), Australia. The survey was conducted at baseline, time point 2 (fourth to sixth month of the implementation year), and time point 4 (tenth to twelfth month). At the same time, the interviews were undertaken at time points 3 (seventh to ninth month) and 4. Participant recruitment occurred simultaneously during these data gathering periods.

The implementation units have a combined capacity of 75 beds, including 10 high‐observation beds. Varying mental health support and treatment are delivered in these units by a multidisciplinary team, including nurses, nurse educators, and peer workers. Each unit has different models of care, peer worker involvement in service, and restrictive practice rates and review approaches. These units operate within a context of high involuntary admissions, with 46% of hospitalisations in acute public mental health services in NSW from 2022 to 2023 being involuntary (AIHW 2025). During the intervention period, and excluding repeated responses for the same individual, most de‐escalation events (58%) in the units involved male inpatients (see Daguman, Yoxall, et al. 2025). The most frequently recorded primary diagnoses were schizophrenia (33%), followed by schizoaffective disorder (13%), and substance‐induced psychotic disorder (13%). There were inpatients without a documented diagnosis (14%).

Ethical approvals for the project were obtained from two Human Research Ethics Committees in a local health district (2023/PID00297–2023/ETH00272) and a university (Approval Number: 2023/069) in NSW, Australia. Written consent for both the surveys and interviews was obtained during the peer‐led survey to streamline the provision of research information and reduce participant burden.

### 2.2 Participants and Recruitment

All survey and interview participants were recruited from the participating acute mental health units during the intervention implementation period, with interview participants recruited from the pool of survey participants. A purposive sampling was employed, with no selection criteria beyond participants being over 18 years of age, having the capacity to provide informed consent, and being willing to use interpretation services if required. The purposive sampling approach was intended to achieve ‘completeness[,] not convergence’ (Madill et al. 2000, 10), in perspectives and experiences across implementation sites. The minimum sample size for the quantitative survey was determined a priori using G*Power 3 (Faul et al. 2007). At least 135 individual survey responses were needed across the intervention sites, with an additional 30% margin for non‐response, to achieve 95% power for detecting a large effect size (*f* = 0.40) across three groups, corresponding to the baseline, time point 2, and time point 4 of the implementation periods. For the interviews, the aim was to recruit 15 participants at the second and fourth time points, guided by estimates of data saturation in qualitative interview studies within multiple sites (Vasileiou et al. 2018).

Individual participants were assessed for their capacity to consent before participating in the survey using a modified version of the University of California, San Diego Brief Assessment of Capacity to Consent (UBACC; Jeste et al. 2007). In accordance with the requirements for administering the UBACC, the assessment was conducted by service staff who held a bachelor's degree or higher. A ‘clearly capable’ response to items 1, 3, 4, 5, 6, and 7 was required for a person to be considered eligible to participate in this study. The tenth item of the UBACC was not used, as it is not applicable in Australia, where a universal public health care system is in place.

### 2.3 Procedures

This research involved a peer‐administered, tablet‐based inpatient survey and follow‐up individual semi‐structured interviews guided by a prompt schedule.

#### 2.3.1 Inpatient Surveys

The survey was administered using a tablet with a touchscreen. The survey began with an introduction and access to the participant information sheet. Participants provided individual consent before completing two psychometric measures: the 20‐item adapted Consumer Evaluation of Mental Health Services Questionnaire (CEO‐MHS; Oades et al. 2010) and the 13‐item Staff Action scale of the Modified Violence Prevention Climate (VPC‐M‐FR; Goulet et al. 2021). Following these measures, participants responded to two researcher‐developed sentence‐completion items, designed to elicit feedback on areas for improvement (‘My experience would have been better if nurses…’) and positive aspects of care (‘The best things about nursing care in this service were…’). Demographic information was collected at the end of the survey.

The CEO‐MHS questionnaire is a patient‐rated measure of experience. It was chosen for its relevance to the current research's geographical context (i.e., developed and tested in NSW) and for the meaningful peer involvement associated with its design and initial evaluation (Oades et al. 2010). For this research, a shortened version of the CEO‐MHS was used, scored identically to the original, comprising a 14‐item Empowerment sub‐scale and a 6‐item negatively scored Dehumanization sub‐scale. Items 5, 11, 14, 17, 38, and 43 of the CEO‐MHS were excluded, because they were deemed irrelevant to the services under study in their current context. Additionally, the Patient Action and Therapeutic Environment scales of the VPC‐M‐FR were not used, as the intervention specifically targeted nurses' relational capabilities. Staff Action scale items 16 and 24 were negatively scored. Higher scores indicate greater satisfaction with service provision or staff actions related to violence prevention. The final look and feel of the Qualtrics‐hosted survey were refined with input from a lived experience practitioner (DO), who had experienced coercive engagement and restrictive practices in acute mental health units.

The survey was offered and administered by peer workers through a protocol to minimise response bias (Oades et al. 2010) and to reflect Safe Steps' value of autonomy. At the end of each survey, participants were invited to elaborate on their experience of the service and of nurses' therapeutic responses to emotional distress, troubling behaviours, and interpersonal conflict through a qualitative interview scheduled approximately 1 month after discharge. Participants who expressed interest in participating provided their contact information and were informed that they could withdraw at any time, even if they had initially agreed to participate.

In this research's dataset, the scores' internal consistency was excellent for both the CEO‐MHS questionnaire (*α* = 0.95, *ω*_*t*_ = 0.95) and the Staff Action scale of the VPC‐M‐FR (*α* = 0.92, *ω*_*t*_ = 0.93). The factorial validity of the scores from the two measures was not assessed due to the sample size, which fell short of even the crude minimum ratio of five participants per one item of a measure (Gorsuch 2014). Nonetheless, as specified a priori, the within‐group comparison was undertaken using the known two‐factor structure of the CEO‐MHS questionnaire and the unidimensional structure of the Staff Action scale.

#### 2.3.2 In‐Depth Semi‐Structured Interviews

The first author interviewed the research participants in English, with the interview mode determined by the participants' preference. It was preplanned that any participant who needed health care interpretation services would be interviewed in their preferred language, including the language they speak at home. Interviews began with reminders about confidentiality, participants' right to pause or withdraw at any time, and the current research aims.

A researcher‐designed, semi‐structured interview schedule was used, comprising six main questions on admission, demographic information, ward experience, person‐nurse relationships, experiences with restrictive practices, and staff actions towards violence prevention. This interview schedule was devised with consideration for the context in which Safe Steps was implemented. Based on the interview participants' responses, relevant follow‐up questions informed by the first author's evaluation and implementation framework were asked, including questions on autonomy (e.g., What does this mean to you?), emergence (e.g., How did it make you feel?), feedback (e.g., What do you think needs to happen next?), and holism (e.g., How does everything fit all in a story?).

Individual interview transcripts were returned to the participants for validation before data analysis (Birt et al. 2016), to ensure that participants' values remained at the centre stage.

### 2.4 Reflexivity

The researcher‐made implementation and evaluation framework served as a reflection of the authors' positions, where understanding events involves eliciting the narratives of people who have lived through the experience. The authors of this research recognised that framing experiences is not something researchers can impose on a person, although a professional perspective remains helpful. Similar to the multiple‐model view that can be applied in complex intervention research (Skivington et al. 2021), the authors acknowledged that various factors would have influenced the context in which the participants' experiences occurred.

A team‐based approach was adopted in the data analysis and interpretation to obtain a comprehensive picture of experiences, rather than relying on inter‐rater agreement (Åsbø et al. 2024). The team of analysts comprised researchers and practitioners from diverse educational, professional, gender, and ethnic backgrounds. A lived experience practitioner (DO) was involved in the data analysis to ensure the perspective of an individual with the lived experience of coercive engagement with mental health services was fully integrated.

### 2.5 Data Analysis

Both quantitative and qualitative approaches to data analysis were applied in this research. For the quantitative strand, changes in unmatched scores from the standardised measures were examined using a one‐way repeated‐measures ANOVA in R (R Core Team 2023) and RStudio (Posit Team 2023). Missing data were imputed for cases with up to 20% missing responses using the mice package (van Buuren and Groothuis‐Oudshoorn 2011), while measures with more than 20% missing items were excluded from the analysis. Missing data were minimal, with scattered item‐level missingness (1–2 cases, ~1%–2%) across both the CEO‐MHS questionnaire and the VPC‐M‐FR measures. One CEO‐MHS case and two VPC‐M‐FR cases were dropped because they had responses of less than 80% of the items, in accordance with the preregistered protocol. A total of 9 values were imputed for both measures. Normality tests indicated non‐normal distributions for all three subscales, supporting the use of non‐parametric analysis for the scores. There was no preregistered covariate modelling for this research. This decision was based on evidence that sample sizes for non‐routine, standardised measures in evaluations of interventions to reduce restrictive practices are often small (see Finch et al. 2021), which are consequently insufficient to support statistical modelling with inverse probability weighting or subgroup analyses.

For the interviews and brief survey commentaries on the best aspects of, and areas for improvement in, nursing care within the implementation units, Braun and Clarke's (2023, 2006) reflexive thematic analysis was applied, encompassing re‐reading transcriptions for familiarity, data organisation, coding and code streamlining, as well as iterative refinement of thematic findings. The first author conducted the analysis in five steps, beginning with (i) re‐reading transcriptions to re‐familiarise with the data. Reflection was undertaken on sections of text that captured interview participants' experiences of nurses' therapeutic responses and support, to recognise themes most emphasised in their accounts. Data were (ii) organised using a spreadsheet, with (iii) codes created. Codes were (iv) streamlined into topical classifications, representing focal and consistent features of experienced care. These codes were (v) further refined and presented to the analysis team for review and final naming of themes. A codebook of themes was maintained during the analysis.

## 3 Results

The following section is divided into two parts: the first reports on the survey outcomes and the second describes the interview themes and survey commentaries.

### 3.1 Inpatient Survey Outcomes

There were 86 survey participants: 39 at baseline, 25 at time point 2 and 22 at time point 4. Over half (*n* = 48) of participants were aged 41 years and above, nearly half identified as female (*n* = 41) and most spoke English at home (*n* = 53). Almost all participants (*n* = 73) were non‐Indigenous and Australian‐born (*n* = 73), with over half identifying as Oceanian (*n* = 54). About 20 had 2 to 3 weeks of inpatient admission length at the time of their survey completion, and a third experienced forced medication (*n* = 28). Additional information is displayed in Table 2.

**TABLE 2:** Survey participant demographic information at the time of survey completion.

Demographic	*n*	% (*n*/*N*; *N* = 86)
Age		
18–30	16	18.60%
31–40	22	25.58%
41 or older	48	55.81%
Gender		
Male	40	46.51%
Female	41	47.67%
Non‐binary/third gender and not otherwise specified (i.e., gender fluid)	2	2.33%
Prefer not to say	3	3.49%
Language spoken at home		
English	53	61.63%
Italian	20	23.26%
Not otherwise specified (including but not limited to Spanish, Te Reo and Turkish)	11	12.79%
Prefer not to say	2	2.33%
Aboriginal or Torres Strait Islander origin		
Yes—Aboriginal or Torres Strait Islander, Aboriginal and Torres Strait Islander	10	11.63%
No	73	84.88%
Prefer not to say	3	3.49%
Country of birth		
Australia	73	84.88%
Canada, Mexico, New Zealand, Philippines, Spain, United Kingdom	10	11.63%
Prefer not to say	3	3.49%
Ethnicity		
Oceanian	54	62.79%
Southern and Eastern European, North African and Middle Eastern, Southeast Asian, People of the Americas	10	11.63%
Others (including but not limited to the United Kingdom)	16	18.60%
Prefer not to say	6	6.98%
Length of Residency in Australia		
6 months to a year, 3–5 years, or more than 5 years	10	11.63%
Prefer not to say or not applicable	76	88.37%
Length of inpatient admission		
Less than a week	17	19.77%
1 week	11	12.79%
< 2 weeks	15	17.44%
2–3 weeks	20	23.26%
4 weeks and beyond	13	15.12%
Prefer not to say	10	11.63%
Restrictive practice experience during inpatient admission (multiple answers possible)		
Seclusion	15	
Physical restraint, mechanical restraint, and other forms (including but not limited to psychological pressure, involuntary admission and transfer to a high observation area)	19	
Forced medication	28	

*Note:* Demographic strata with fewer than 10 samples were combined with adjacent categories to preserve anonymity.

**FIGURE 1:**
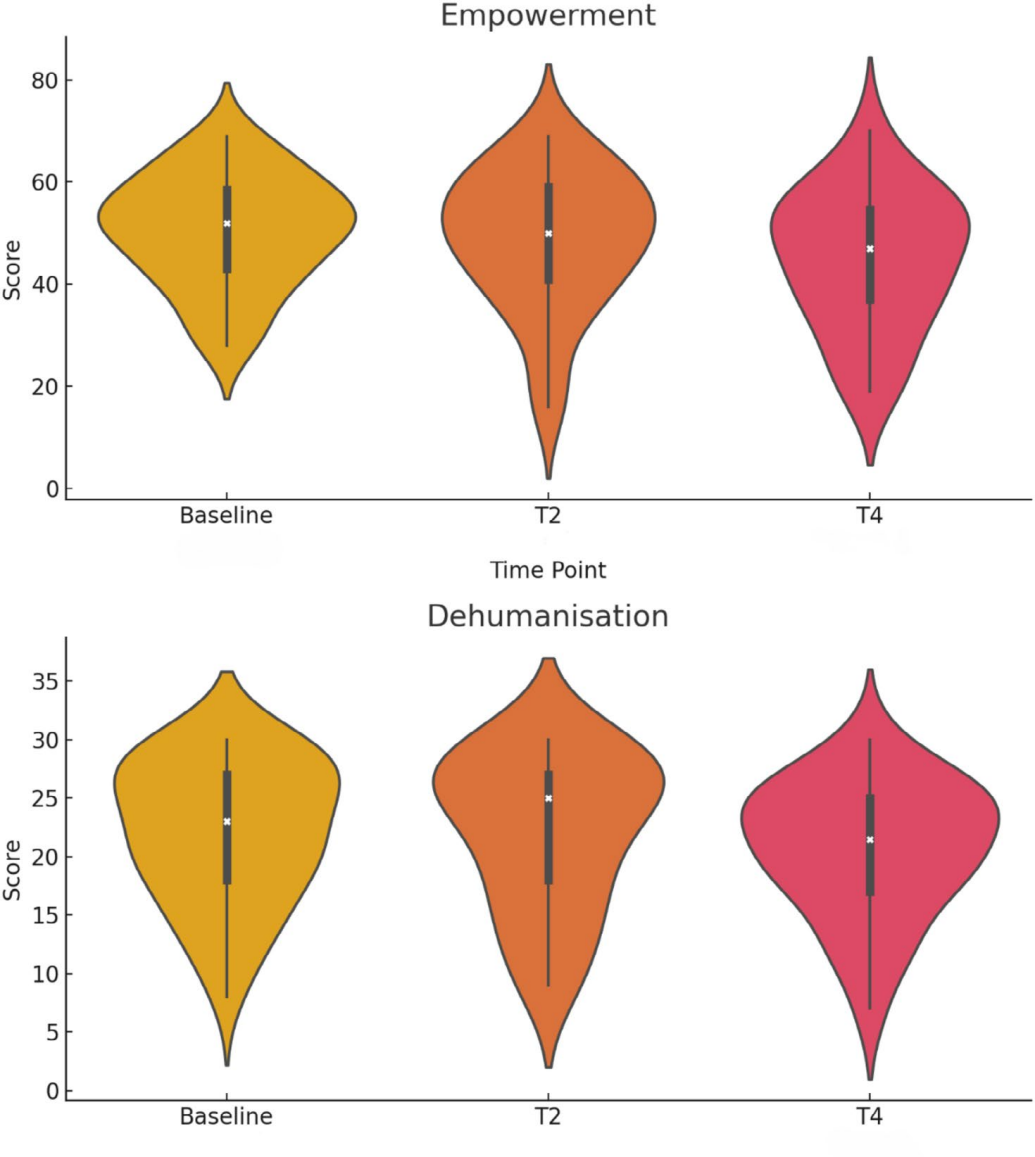
Violin plots for scores on Empowerment (top plot) and Dehumanization (bottom plot) subscales of the Consumer Evaluation of Mental Health Services Questionnaire.

**FIGURE 2:**
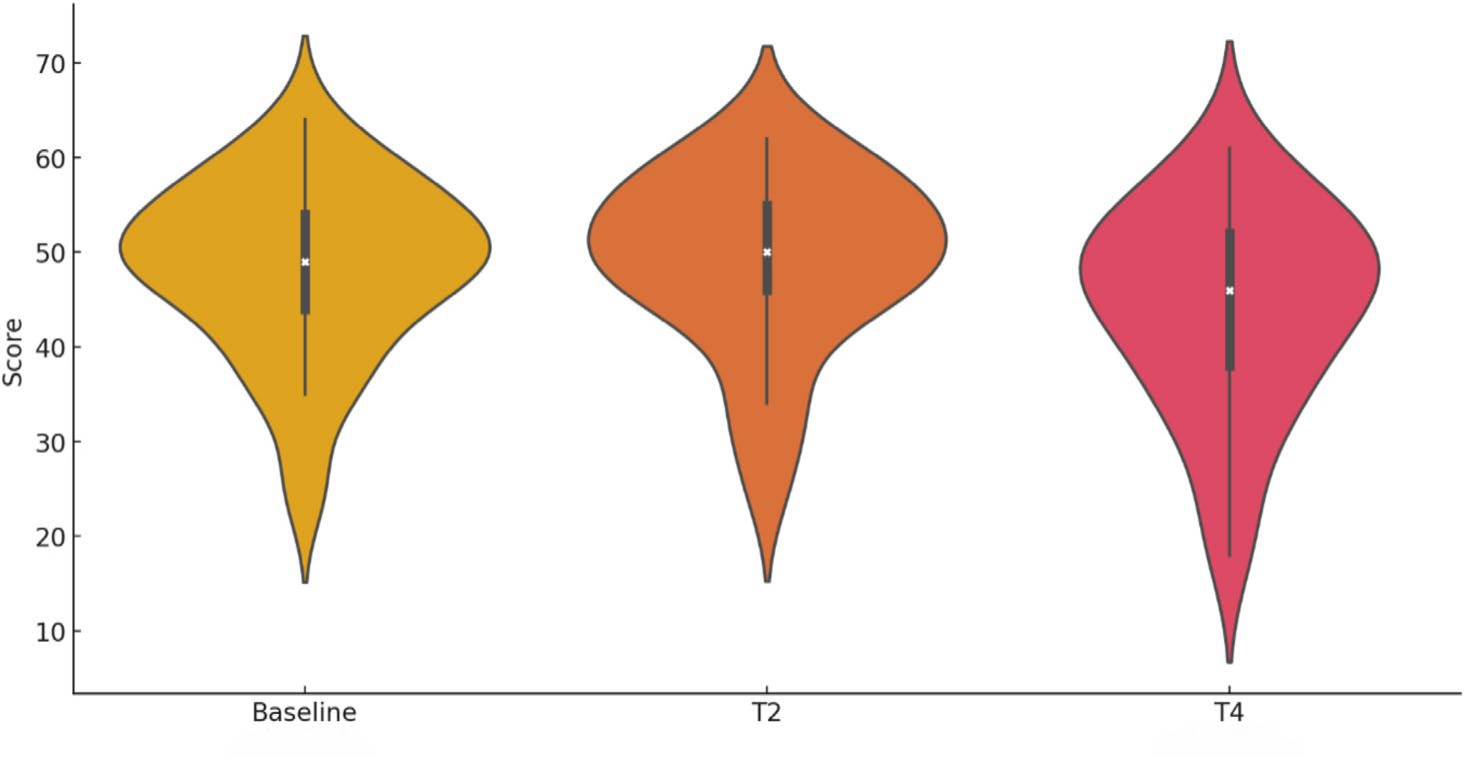
Violin plot for scores on staff action towards violence prevention of the Modified Violence Prevention Climate Scale.

Kruskal–Wallis tests demonstrated no statistically significant differences across time points for Empowerment, Dehumanization, or the Staff Action sub‐scales (all *p* > 0.05; see Table 3), and post hoc Dunn tests with a Bonferroni adjustment likewise found no significant pairwise differences. Median values for Empowerment declined across time points. In contrast, medians for Dehumanization and Staff Action showed small fluctuations (see Figures 1 and 2). Given the null effect noted, the hypothesis for this research was considered unsupported.

**TABLE 3:** Kruskal–Wallis test results and descriptive statistics across time points.

Measure, time point and test results	Mdn	*M*	SD	IQR	*d* (Baseline versus Time Point)
Empowerment					
Baseline	52	50.44	10.84	15.5	—
Time 2	50	48.76	13.36	18	0.14 [−0.36, 0.64]
Time 4	47	44.71	13.56	17.5	0.48 [−0.03, 0.99]
*χ* ^2^(2) = 2.72, *p* = 0.26					
Dehumanization					
Baseline	23	22.10	6.05	9	—
Time 2	25	22.08	6.65	9	≈0 [−0.50, 0.50]
Time 4	21.50	20.67	5.67	8	0.24 [−0.26, 0.75]
*χ* ^2^(2) = 1.47, *p* = 0.48					
Staff actions towards violence prevention					
Baseline	49	48.30	9.12	10	—
Time 2	50	49.04	9.28	9	−0.08 [−0.58, 0.42]
Time 4	46	44.04	10.75	14	0.43 [−0.08, 0.94]
*χ* ^2^(2) = 3.66, *p* = 0.16					

### 3.2 In‐Depth Semi‐Structured Interview and Survey Commentary Findings

Nine of the 27 participants who initially indicated a willingness to be interviewed ultimately completed an interview; the remaining participants were lost to contact. Two participants were interviewed via online video teleconferencing, six by phone, and one in person. The interviews lasted between 23 and 43 min, with a median of 33 min. Only two implementation sites were represented in the interviews, with one site contributing three participants and the other six. Half of the participants were in their thirties. All identified as non‐Indigenous, spoke English at home, and nearly all were born in Australia. Many had multiple admissions to an acute mental health inpatient unit. Given the small sample size (*n* < 10), a more detailed demographic breakdown was not provided to preserve participant anonymity.

**FIGURE 3:**
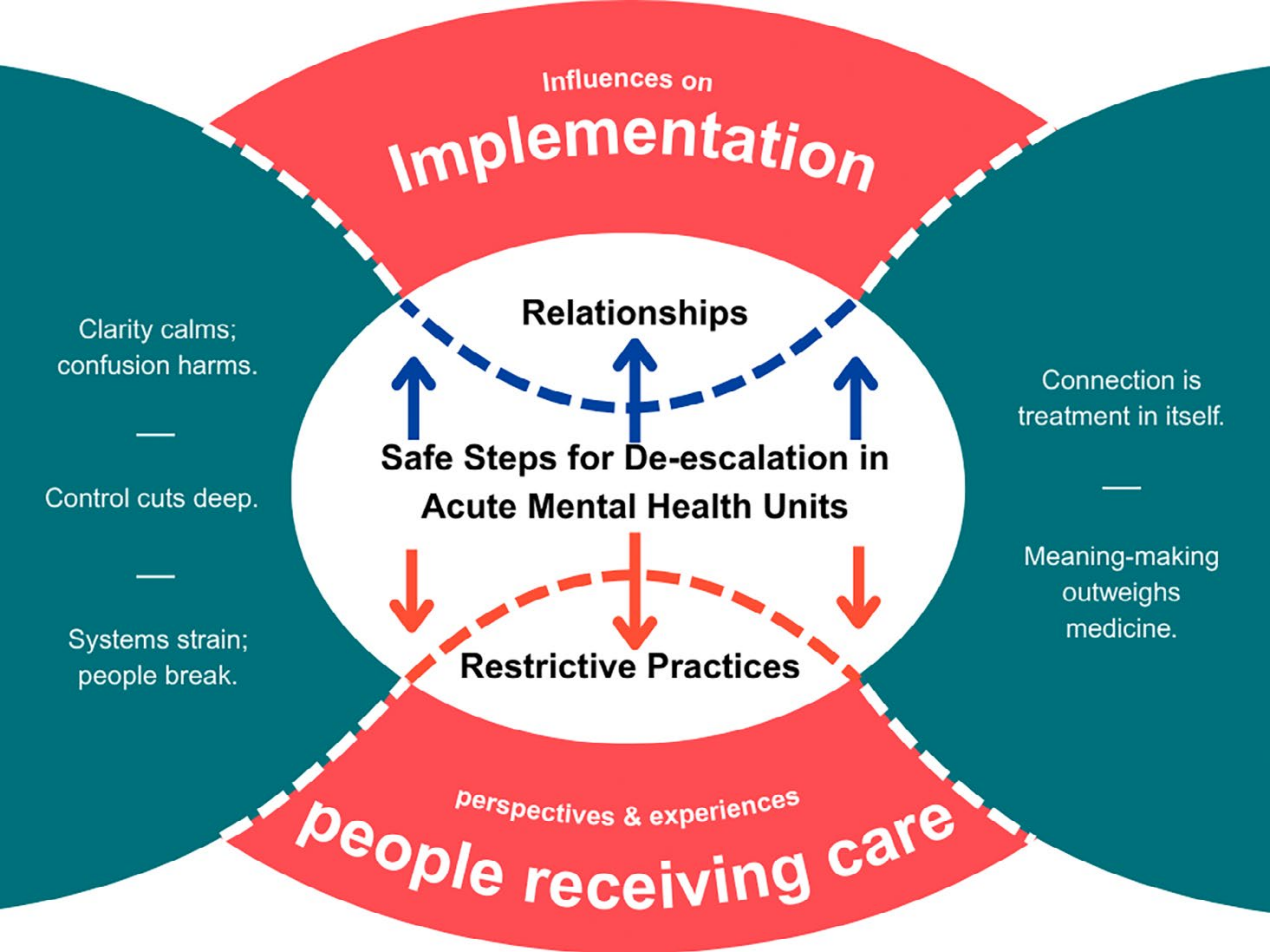
Influences on successful intervention implementation from the perspectives and experiences of inpatient and discharged patient participants. Many visual components in this image, such as colours, shapes, lines, text contents, and text placements, are adapted from an image published in open‐access research (Daguman, Taylor, Flowers, Owen, et al. 2025).

Figure 3 presents the five superordinate themes identified through the reflexive thematic analysis of participants' perspectives and experiences in acute mental health settings from both interviews and survey commentaries: (i) *Clarity calms; confusion harms*, (ii) *Control cuts deep*, (iii) *Systems strain; people break*, (iv) *Connection is treatment in itself,* and (v) *Meaning‐making outweighs medicine*.

#### 3.2.1 Clarity Calms; Confusion Harms

Clear and honest communication was considered important to perceptions of safety, trust, and cooperation during inpatient care. Participants consistently depicted how uncertainty or inconsistent information regarding medications and unit procedures, including their admission and discharge plans, heightened their sense of fear, resistance, and emotional distress. In contrast, small acts of clarification, as in ‘telling…what the medicine was’ (T2A), were considered helpful in making participants feel at ease, more cooperative, and less likely to escalate.

Discussions around medication were a common source of tension. Some participants recollected refusing medication because they were not informed of what they were being given, with one exclaiming, ‘I was not going to take anything until I knew what it was and the dosage’ (T2D). Others described situations where nurses had to ‘go back and have a look’ (T4C) before confirming what was being administered.

Confusion was noted to arise from communication gaps between services within the hospitals, particularly between emergency departments and the acute mental health unit, regarding key information on care recipients' preferences, histories, and backgrounds, as well as treatment and care processes. Several survey and interview participants disclosed being told one thing by the emergency department and undergoing something utterly different in the mental health unit. One exclaimed, ‘Whatever doctor you have got coming from emergency, they are not passing that information on to these guys over [the acute mental health inpatient unit]’ (T2B). Another quipped, ‘What was said [at the emergency department] … was not what was followed [in the mental health inpatient unit]’ (T2D).

Discharge planning brought about its own frustrations, with repetitive, unkept promises that led to interview participants' feelings of disappointment and mistrust. One participant pointed out that they were ‘promised at least a dozen times when [they] would get out’ (T2F). At the same time, another stressed the need to ‘have those open communications with families’ (T2E). Other participants called for genuine conversations in which ‘professionals and individuals receiving care… sit down and actually have an open and honest chat’ (T4B).

#### 3.2.2 Control Cuts Deep

Participants described experiences of coercion, encompassing not only the formal type, such as forced injections, seclusion, and locked wards, but also the more informal exercise of control through rigid rule enforcement, threatened punishment, and the dismissal of their own perspectives. Many visualised nurses as powerless intermediaries, tasked with carrying out psychiatrists' decisions with little room for advocacy or negotiation.

Many accounts focused on overt restrictive measures. One participant recounted being ‘held down and jabbed… violated the only space’ (T2F) they had, while another described being told to take medication that ‘made [them] vomit every time [they] had it… or [else they were] going to have to be given a needle’ (T4A). These moments of formal coercion were often remembered in visceral, embodied terms, with some participants having experienced extrapyramidal side effects after being discharged, including but not limited to body tremors, balance issues, and rigidity. Participants who experienced these side effects considered them as blocks to their ability to return to their work and their everyday lives.

Interviews and survey commentaries also revealed that informal coercion was pervasive. Limitations on people's movement or contact with people and the world from outside the inpatient unit were likened to ‘being in jail’ (T4A), in a ‘zoo’ (ST119), or in ‘Hotel California’ (ST217), where ostensibly negligible rules, such as cigarette smoking breaks, were seen to cause conflict. For some participants, the presence of police in a mental health unit was ‘very triggering’ (T4B). Others felt punitive approaches undermined their personal recovery: ‘Instead of rewarding the patient for doing good things, you punish the patient for doing the bad things… research has shown it does not work’ (T2F).

Imbalances in power between psychiatrists and nurses were frequently brought to the fore. Many participants felt that ‘nurses have no power… if a psychiatrist says something, the psychiatrist says’ (T4A) and asks it to be implemented. Others described psychiatrists as arriving with ‘a premeditated diagnosis’ (T4B) or being ‘stuck on diagnosis’ (ST217). Psychiatrists were further perceived as dismissive of people's alternative explanations for distress, which may have nothing to do with clinical models for meaning‐making. As one person recounted, ‘the doctor… was pushing too hard for psychosis and would not listen to my thoughts about it’ (T2A). Nurses, in turn, were often positioned as the enforcers of unpopular rules, ‘getting the brunt of it’ (T2C).

The interview participants considered the psychological toll of these power arrangements significant. Some participants feared either being ‘turned into a zombie’ (T2D) or that ‘if [they] do not start taking medication, [they] will be [in the unit] for a lot longer’ (T4B).

#### 3.2.3 Systems Strain; People Break

Participants described how systemic issues, including understaffing, staff burnout, and neglected infrastructure, were barriers to safe and therapeutic care. Many participants associated perceived nurse fatigue with the risk of emotional unavailability. Participants reflected on how infrastructure neglect can signal a lack of care and investment in the acute mental health unit. Participants were not only critical, they also offered thoughtful and ‘doable’ suggestions for improving the system.

Understaffing was a repeated issue highlighted by survey and interview participants. Despite this issue, some participants noted that nurses ‘try and make the time for each patient’ (T2C). On the other hand, some participants were concerned that nurses ‘do not remember conversations they have had the next day’ (T2E). High inpatient loads were considered as leaving nurses ‘always [having] somewhere to run to and something to do’ (T2D). A survey participant suggested that ‘if [nurses] had [fewer] patients…there could be more interaction between [a] nurse and patient, so that [patients would] fe[el] more heard, and the nurses would also be not so rushed’ (S034).

Frustrations regarding the poor condition of the inpatient units' physical infrastructure were also expressed during interviews and surveys. One participant (T2D) described ‘a bathroom light that flashed… all the time’ and how it took time for the inpatient unit to get the food refrigerator fixed. T2D further reflected that these issues exemplify the ‘over‐bureaucratisation’ in mental health services, where there is ‘too much effort on the wrong things and not enough effort on the things that are just obvious,’ as in focusing on ‘a fridge [that] is not [profanity redacted] unsafe’ instead of the food in it ‘that has gone off.’ Another survey participant similarly stated, ‘There could be better facilities for patients’ (S219).

Several survey and interview participants had put forward constructive ideas for change. One suggested that instead of multiple nurses ‘just sitting there watching like “Big Brother” and taking notes’ (T2F), a position could be replaced with a behavioural therapist. Calls for greater cultural and social inclusion were also evident, particularly regarding representation and responsiveness for Indigenous inpatients. Some enjoined staff to give greater ‘attention to detail, respect, culture, [and] sexual orientation’ (S233). Another survey participant commented, ‘Nursing staff are underpaid, and should have their own car park’ (ST122).

#### 3.2.4 Connection Is Treatment in Itself

Survey and interview participants had focused on small relational gestures of kindness, humour, and shared background as the elements that made a difference to their acute care experience. Participants specifically narrated how some nurses, cleaners, and health security assistants (HSAs), who treated them as individuals, helped restore their dignity, eased their loneliness, and created micro‐moments of healing.

Gentle, non‐authoritarian approaches were consistently illustrated as nurturing a sense of safety and trust. One participant mentioned, ‘[nurses] did not jump down my throat… they just thought, “we will use gentle hands with you, mate,” and just sort of guide me in the right direction’ (T2C). Others spoke of nurses going above and beyond, so the inpatients would ‘always leave feeling like [they are] somebody’ (T4B), or connecting more easily with nurses, who ‘told a little bit of a story about themselves… [and] spoke as an equal’ (T2D).

Interview participants also valued it when nurses saw them as a whole person, rather than a mental distress diagnosis. ‘Some just treated us as people without an illness’ (T2E), one participant noted. Small gestures, such as offering a warm drink, helping with personal hygiene, or ‘bother[ing] to find out who [they were]’ (S122), were recalled as helpful in instilling a sense of inclusion and worth. One commented, ‘everybody [the nurses] just wants to feel like they [the inpatients] belong’ (T4B). In the same breadth, a notable observation in both surveys and interviews was that there were several written and spoken mentions and recalls of specific nurses' names with whom participants had built rapport, regardless of whether the interaction spanned a sustained period or only a brief encounter, such as a five‐ to ten‐minute conversation.

#### 3.2.5 Meaning‐Making Outweighs Medicine

Participants expressed a hunger for meaningful engagement beyond medication, including opportunities for creative self‐expression, learning, movement, or peer contact, that could restore their sense of autonomy, identity, and emotional connection. Many participants qualified that their inpatient admission days were shaped by boredom, locked resources, or passive containment, where treatment was reduced to adherence to medication. When opportunities for meaningful engagement arose, whether self‐organised (such as asking nurses to unlock art cupboards or helping peers) or staff‐supported, participants indicated that these rare moments were purposeful for them.

The absence of structured activities left many feeling unstimulated. ‘One activity in three weeks’ (T2B) was how one participant deduced their stay, while another remarked, ‘There was no activity officer… they are all bored’ (T2F). Even when art therapy was nominally available, they ‘had to fight to get art stuff’ (T2E).

Some participants sought to fill this gap on their own. One interview participant recounted that ‘all the cupboards to the art[s] and craft[s] were locked up’ (T2F) until they began asking for them to be opened, eventually bringing out board games. Others took on informal helping roles, with one stating, ‘I kind of acted as a peer worker, and they let me do that’ (T2E), or created meaning in small personal contributions like ‘offering water in the garden… gave [them] purpose and something to do’ (T2D).

When it came to mental health treatment, some participants frequently cited that risk assessment and ‘medication alone does not solve things’ (T4C). Interventions that address underlying issues and support personal recovery, such as access to therapy, psychologists, or counsellors, could be helpful. A participant suggested that having access to activities like ‘meditation, yoga, [and] understanding anxiety’ (T4A) could improve their own recovery and even reduce the length of high‐dependency stays. Others wanted staff to ‘understand treatment and the difference between mental health [issues] and drug use’ (S117).

## 4 Discussion

Across the three time points, there were no statistically significant changes in standardised measures of perceived empowerment, dehumanisation, and staff actions on violence prevention. However, median scores were mainly on the favourable side of the measures' ranges. These findings do not suggest that the Safe Steps implementation and the outcome measurement process were not rigorous. Instead, they could be more reflective of the instruments' *in*validity and *in*sensitivity to change over time, which were already pointed out in a systematic review of 23 studies on the content and outcomes of interventions to alleviate social climate in acute mental health units (Dickens et al. 2022). In this research, estimating the fit between the scores and the known factor structure of the measures was not possible, indicating that the scores obtained may not reflect what the measures were intended to assess. While the scores' internal consistencies were excellent, inference‐making requires broader psychometric validity support, as these properties pertain to the quality of the scores in a given context, rather than to the instrument itself, whose original validity has already been established using a different dataset (Daguman and Taylor 2025). Furthermore, similar to the measure of nurses' professional quality of life in the larger study evaluating the Safe Steps implementation (Daguman et al. 2026b), service experience and ward climate could be considered as distal outcomes that are more heavily influenced by peripheral, non‐intervention implementation factors (Brenner et al. 1995). That is, other factors could have introduced biasing paths between the Safe Steps implementation and outcome measures.

The lack of change observed in the standardised measures is similar to what has been reported for Safewards on measures of ward climate (Bowers et al. 2015; Dickens et al. 2020) and for a psychoeducation and preventive monitoring programme across many domains of empowerment measures (Lay et al. 2015). These findings are consistent, in part, with the thematic findings from the interviews and survey commentaries in this research, which suggest that while some participants described subtle, positive aspects of support during admission, others relayed coercive experiences. Contrasting these null effects are the reduced restrictive practice events observed in the larger Safe Steps study at months three and nine of the implementation year (Daguman, Yoxall, et al. 2025). This divergence in impact finds semblance in other mental health intervention evaluations (e.g., one‐to‐one peer support) that have influenced measures of psychosocial outcomes (e.g., empowerment, therapeutic relationships) without producing measurable changes in clinical outcomes (e.g., symptomatology and hospitalisation; White et al. 2020).

Five qualitative themes emerged from the interviews and survey commentaries; three reflected issues within the inpatient units, and two identified perceived solutions to address these issues, at least in part. These themes suggest that participants had a wider aperture for telling their rendition of their stories. Although the participants did share personal and observed experiences of nurses' de‐escalation practices, they were not centred on the technical aspects of the Safe Steps. This thematic emphasis may be partly explained by the fact that the intervention was intended to be, or has been, embedded in routine care by nurses, who are the direct target of the intervention. A similar emphasis was evident in a discursive paper in Victoria, Australia, critiquing the Safewards model, where the authors with lived experience of using inpatient services moved from issues of reducing restrictive practices in their analysis towards broader concepts of safety, ward culture, and potential harms in inpatient care (Kennedy et al. 2019).

As expressed in the first three of the five thematic findings, there was a co‐occurrence of coercion, process and communication gaps, and systemic strain in the acute mental health units where Safe Steps was implemented. This co‐occurrence depicts acute mental health inpatient units as circuits of power (Hutchinson and Jackson 2015; Hutchinson et al. 2010) in which people receiving care are socialised into the institution's professional accountability‐focused, control‐oriented, and (seemingly) value‐free logic that resists change. Through repeated inpatient admissions and exposure to formal and informal coercion, the participants learned that compliance is rewarded, harmful risks and positive risk‐taking are both evaded, dissent is perilous, and clinical authority is seldom open to negotiation. These experiences reflect those found in a recent meta‐ethnography of twenty‐seven qualitative studies in adult mental health units, in which restrictive practices' function was described as an abuse of power, with poor communication further embedding mistrust between people receiving care and staff (Griffin et al. 2025).

The opacity of decision‐making, where psychiatrists are positioned as ultimate arbiters of what is (has been and will be) happening for people receiving care, and nurses are constrained instruments of these arbiters, reinforces a hierarchy that privileges specific voices and forms of knowledge while sidelining others (Lakeman 2000; Roy 2023). This privileging of certain voices supports the idea that people with mental distress are unjustly considered untrustworthy sources of testimonies (Lakeman 2010), who do not own their minds. Nevertheless, the qualitative themes from nurse focus group discussions, the statistically significant paired measures of nurses' professional quality of life from nurses surveys (Daguman et al. 2026b), and the commentaries on the events preceding restrictive practices from nurses' de‐escalation logs (Daguman et al. 2026a) in the larger study in which this research is situated corroborate many testimonies shared by the survey and interview participants, including those about chronic understaffing, nurses' burnout, and neglect of physical infrastructure. These corroborated themes provide support to people receiving care as a credible source of testimonies, while tacitly communicating the priorities and resource allocation decisions of the acute inpatient unit's service management and the wider organisation's leaders who administer them. Recent support for similar institutional barriers has been found in a qualitative study of various Norwegian mental health services, in which participants highlighted that such barriers hindered opportunities to form and maintain therapeutic relationships (Iversen et al. 2025).

The last two thematic findings on connection and meaning‐making challenge the development of a detached monoculture in which mental health support and treatment for people with mental distress who have everyday life problems revolve around specialised interventions (Slade 2012). Instead, these themes elevate participants' valuation of ‘everydayness’ (Skatvedt 2017, 398), where care grounded in seemingly ‘empty’ human gestures and opportunities that mirror life outside the acute inpatient unit are viewed as central to personal recovery. These recovery‐promoting gestures have been illustrated in the two themes and are said to evoke ‘Kama Muta,’ a state of movement that strengthens bonds and nurtures engagement in care (Alessandrini 2024). These gestures further suggest that a subset of nurses in the implementation sites employed high‐quality mental health practices. Lakeman's (2012) survey of thirty experienced mental health nurses in Ireland provides support to this statement. He found that small, caring gestures are integral to ‘good mental health nursing,’ where interventions are woven into everyday, close‐range interactions, rather than delivered in structured sessions or designated therapy settings. On the other hand, the participants' specific valuing of creative, social, and skill‐building activities aligns with the literature, which states that aesthetic and participatory practices can nurture agency, facilitate meaning‐making, and foster mutual recognition and belonging in mental health recovery (Damsgaard et al. 2025). Indeed, everyday ‘doings’ are more than just practical acts (Friesinger et al. 2025); they hold meaning that affirms personhood, strengthens existing relationships, and fosters recovery that is not solely remedied by medication.

### 4.1 Strengths, Limitations and Future Research Directions

A key strength of this research is its involvement of lived experience practitioners, specifically people who experienced coercive engagement with acute mental health services, in various aspects of the intervention research. This strength aligns with calls for greater inclusion of lived experience perspectives in coercion research (Gooding et al. 2020). Also, the involvement of Indigenous peoples in the co‐development of an intervention component, the capacity‐to‐consent in research assessments, the peer‐led survey administration, the qualitative data validation, the chosen standardised measures developed in NSW with input from people with lived experience of mental health service use, and the framework for evaluating the Safe Steps are features that set this research apart from other studies in the field, where such participatory (Finch et al. 2021) and theorised approaches (Lantta et al. 2023) are often absent.

This research is not without limitations. First, survey and interview sample sizes were smaller than planned, with interview participants representing only two of the three implementation sites. This limitation restricts the generalisability of both quantitative findings and qualitative insights. However, it is important to note that the process of offering and gathering surveys and interviews was itself a complex undertaking. The authors and lived experience practitioners considered this process to be influenced by the existing rapport or relationship, or the lack thereof, between the participant recruiter or the capacity‐to‐consent in research assessor and the potential participant, even when the lived experience practitioner was physically present, allaying the expressed worries of the targeted participant surrounding research participation. Second, the absence of randomisation and the inability to apply statistical adjustments to account for confounding further constrain the strength of the inferences drawn from the research.

The frequent recall and mention of individual nurses' names during surveys and interviews suggest that notable therapeutic relationships may have developed during the implementation of the intervention. Therefore, measures of the quality of the therapeutic relationship between the person and the nurse with whom the person had the most contact during their inpatient admission could be considered a primary outcome in further evaluations of the Safe Steps and routine inpatient care. This targeted measure could help link individualised relational experiences to overall perceptions of care, and it may be more sensitive to the intervention's relational focus. In addition, routinely‐collected measures of experienced service could be considered an alternative to the purposefully selected measures used in this evaluation, as they may reduce participant burden, detection, and performance bias, while improving response rates. Lastly, measures of informal coercion will need to be considered for evaluating restrictive practice‐reduction interventions, as this research demonstrated that coercion was not limited to those legally‐regulated.

### 4.2 Relevance for Clinical Practice

This research elevates the central role of nurses in shaping everyday service experiences and in supporting the personal recovery of people receiving care. Nurses in acute mental health units can help people receiving care in their quest for meaning in life through small, humane gestures of care, transparent communication of treatment processes and medication (which may or may not matter for the person receiving care), and organising inspiring and creative outlets for individuals to express themselves, support their fellow inpatients, and discover their potentials as whole human beings.

### 4.3 Lived Experience Commentary (Dane Owen)

I agree with the participants' sentiments, views, and experiences, particularly those expressed in the qualitative data. Coercion remains a pressing issue, and I do not believe that restrictive practices should be routine; they may be appropriate only in very rare circumstances, perhaps around 1% of the time, such as in cases of imminent risk of harm to oneself or to others, including staff, peers, and fellow patients.

Regarding communication gaps, I believe it is vital for mental health clinicians to be transparent and relational and to talk *with*, rather than *at*, the person. While it is true that the mental health workforce is under‐resourced, I share the perspective of many interview participants that even a few minutes can be enough to make meaningful connections. As a respected colleague of mine in peer work once summed it up: ‘If you are not on the floor, you do not have rapport.’ I also concur that acute mental health services often focus disproportionately on medication, rather than offering more holistic support and treatment. Medication can certainly be helpful, but it is too often used as a band‐aid or one‐size‐fits‐all solution, sometimes without full consideration of the person's physical and psychosocial health. For me, considering the whole person means enabling people to actively contribute to their clinical treatment, particularly by having a choice in their own psychopharmacological care, which can be empowering and validating. Lastly, inspiration and hope for recovery often arise from unlikely places. I believe that there are unsung heroes within inpatient units, including the group activity coordinators, cleaners, HSAs, and assistants‐in‐nursing, who help inpatients get through boredom and make sense of their admission.

The experience of being an active researcher in this endeavour has been both valuable and empowering for me. Hearing people's authentic voices has been enlightening; it has given me a deeper sense of understanding and shifted my perspective on de‐escalation, particularly on how to respond to people in distress or crisis and on the importance of being collaborative in escalated situations. In reflecting on these lessons, I am reminded of lyrics I once wrote:

*I can't understand*.
*Nothings turned out to plan*.
*What the hell went wrong?*
*It's all come undone*.
*And I say, ‘what about?’*
*Got to see through the clouds*.
*No one hears. So, I scream and shout*.
*When I'm alone*.

## Author Contributions

Esario Daguman IV: Conceptualisation, methodology, software, formal analysis, investigation, resources, validation, data curation, writing – original draft, and visualisation. Dane Owen: Methodology, investigation, and resources. Jacqui Yoxall: Resources and writing – review and editing. Richard Lakeman: Resources and writing – review and editing. Marie Hutchinson: Conceptualisation, methodology, resources, writing – review and editing, funding acquisition, and supervision.

## Funding

This research is part of a larger study supported by Southern Cross University and the Translational Research Grant Scheme from the NSW Office for Health and Medical Research.

## Ethics Statement

Ethics approval was obtained from two Human Research Ethics Committees in a local health district and a university in New South Wales, Australia. Consent to participate in research was obtained from the participants of this research.

## Consent

The authors have nothing to report.

## Conflicts of Interest

Esario IV Daguman is supported by a PhD scholarship jointly funded by Southern Cross University and the Translational Research Grant Scheme of the NSW Office for Health and Medical Research. One funding body provided feedback that was considered in the initial protocol development to strengthen the study’s scientific merit. Neither organisation contributed to the original conceptualisation or to the conduct, analyses, reporting, interpretation, and writing of this research. The views expressed in the Introduction and Discussion sections of this research are those of the authors and do not necessarily represent those of the funding bodies, the organisational affiliations of the authors, nor those of the research and implementation partners of the larger project in which this research is situated.

## Data Availability

The datasets gathered, analysed, and interpreted for this research are not publicly available, due to conditions in the ethical approvals obtained.

## Endnotes

^1^
People who use acute mental health services are human beings first (Anthony 2004). In this research, the authors used the terms ‘people,’ ‘persons,’ or ‘individuals’ to acknowledge their vocation to be wholly human. In contrast, many survey and interview interactions and responses suggest that participants were unfamiliar with, or chose not to identify with, the commonly used term in public mental health services across NSW, Australia (i.e., ‘consumers’). The authors acknowledge the ongoing debate over preferred terminologies in mental health research, and terms like ‘patient’ have been retained where they were used by the interview and survey participants themselves.
